# Blood routine test in mild and common 2019 coronavirus (COVID-19) patients

**DOI:** 10.1042/BSR20200817

**Published:** 2020-08-07

**Authors:** Dodji Kossi Djakpo, Zhiquan Wang, Rong Zhang, Xin Chen, Peng Chen, Malyn Martha Lilac Ketisha Antoine

**Affiliations:** 1 Department of Cardiology, Zhongnan Hospital of Wuhan University, Wuhan 430071, Hubei, China; 2 Department of Neurology, Hubei Aerospace Hospital, Xiaogan 432000, Hubei, China; 3 Department of Respiratory Medicine, Hubei Aerospace Hospital, Xiaogan 432000, Hubei, China; 4 Department of Endocrinology, Zhongnan Hospital of Wuhan University, Wuhan 430071, Hubei, China

**Keywords:** CBC, COVID-19, MERS-CoV, SARS-CoV

## Abstract

The new 2019 coronavirus disease (COVID-19), according to the World Health Organization (WHO), has been characterized as a pandemic. As more is being discovered about this virus, we aim to report findings of the complete blood count (CBC) of COVID-19 patients. This would serve in providing physicians with important knowledge on the changes that can be expected from the CBC of mild and normal COVID-19 patients. A total of 208 mild and common patients were admitted at the Dongnan Hospital located in the city of Xiaogan, Hubei, China. The CBCs of these patients, following a confirmed diagnosis of COVID-19, were retrospectively analyzed and a significant *P*<0.05 was found after a full statistical analysis was conducted using the Statistical Package for the Social Sciences (IBM SPSS). CBC analysis revealed changes in the levels of red blood cells (RBCs), hemoglobin (HGB), hematocrit (HCT), mean corpuscular volume (MCV), and C-reactive protein (CRP). Clinicians should expect similar findings when dealing with the new COVID-19.

## Background

The novel coronavirus outbreak, which was first reported in Wuhan, China has now spread worldwide and is characterized by the World Health Organization (WHO) as a pandemic with Europe now being considered a new epicenter of the virus [1,2]. On 14 March 2020, global reports noted a total of 142539 confirmed cases (9769 new) and 5393 deaths (438 new), while in China 81021 (18 new) cases were confirmed with 3194 (14 new) deaths [3]. The 2019 coronavirus disease (COVID-19) shares similar epidemiological characteristics with the severe acute respiratory syndrome coronavirus (SARS-CoV) and the Middle East respiratory syndrome coronavirus (MERS-CoV). However, their mortality rates differ significantly as COVID-19 presents a lower mortality rate (2.08%) as compared with SARS-CoV (10.87%) and MERS-CoV (34.77%). Despite this fact, the COVID-19 is more contagious [4]. Regardless of the differences noted, these diseases manifest similarly with a cough and fever [5,6]. Laboratory findings, especially complete blood counts (CBCs), play an essential role when dealing with infectious diseases. As the investigation into the novel COVID-19 continues to grow, we aim to report the CBC findings of the new viral disease hoping that this report will provide useful information to all physicians. This report aims at investigating the significant changes observed in the CBC of mild and common COVID-19 patients.

## Materials and methods

This is a retrospective observational study. We retrospectively collected and analyzed data from 208 mild and common COVID-19 patients admitted to the Dongnan Hospital located in Xiaogan, Hubei, China. Data were collected from January to March 2020. Information was drawn from patients’ electronic medical records. All patients were confirmed as positive for COVID-19 based on a history of exposure to the virus, clinical manifestations, lungs computed tomography (CT scan), and pharyngeal swab specimen’s nucleic acid amplification test by reverse transcription-polymerase chain reaction (RT-PCR) according to the fifth edition of diagnosis and treatment plan. Patients with negative results for COVID-19 and patients presenting with severe and critical conditions were excluded from the present study. All procedures performed in the present study were in accordance with the ethical standards of the national research committee and with the 1964 Helsinki Declaration and its later amendments. Informed consent was obtained from all patients involved in the present study.

### Statistical analysis

The first whole CBCs of confirmed COVID-19 patients were analyzed. Statistical analysis was assessed by the Statistical Package for the Social Sciences (IBM SPSS 22). The normal distribution measurement was expressed by mean ± standard deviation. The independent *t* test was used to compare the means of two groups and the Mann–Whitney U test was performed to compare differences between two independent groups. We considered *P*<0.05 as statistically significant.

## Results

Overall, data of 208 mild and common confirmed cases as COVID-19 was analyzed. The median age of subjects used in the present study was 50 years. Data from 107 (51.4%) males and 101(48.6%) females were used for analysis. All patients’ characteristics are shown in Table 1. Some patients were admitted with comorbidities which included: hypertension 41 (19.7%), respiratory diseases 16 (7.7%), diabetes mellitus 13 (6.3%), and coronary heart disease 11 (5.3%).

**Table 1:** Baseline characteristics of all included patients

Characteristics	*n* (%) or Median ± Std. deviation
Age	50 ± 17.475
Gender - *n* (%)	
Female	101 (48.6)
Male	107 (51.4)
History and comorbidity - *n* (%)	
Respiratory diseases	16 (7.7)
Hypertension	41 (19.7)
Coronary heart disease	11 (5.3)
Diabetes mellitus	13 (6.3)
Alcohol	9 (4.3)
Smoking	12 (5.8)
Symptoms - *n* (%)	
Asymptomatic	28 (13.5%)
Symptomatic	180 (86.5%)

The laboratory findings (Table 2) included white blood cells (WBCs), red blood cells (RBCs), hemoglobin (HGB), platelets (PLTs), neutrophils, lymphocytes, monocytes, eosinophils, basophils, hematocrit (HCT), mean corpuscular volume (MCV), mean corpuscular HGB (MCH), MCH concentration (MCHC), platelet distribution width (PDW), and C-reactive protein (CRP) counts. Fever (70.2%), cough (55.3%), dyspnea (27.4%), and fatigue (22.6%) were the predominant symptoms in all patients (Table 3). One hundred and eighty (86.5%) patients were symptomatic.

**Table 2:** CBC results of all patients

Laboratory parameters	Normal ranges	Mean ± Std. deviation
WBC (×10^9^/l)	3.5–9.5	5.76 ± 2.656
RBC (×10^12^/l)	4.3–5.8	4.24 ± 0.619
HGB (g/l)	130–175	131 ± 18.255
PLTs (%)	125–350	190.52 ± 79.492
Neutrophils (×10^9^/l)	1.8–6.3	3.76 ± 2.450
Lymphocytes (×10^9^/l)	1.1–3.2	1.47 ± 0.622
Monocytes (×10^9^/l)	0.1–0.6	0.4 ± 1.99
Eosinophils (×10^9^/l)	0.02–0.52	0.09 ± 0.118
Basophils (×10^9^/l)	0–0.06	0.03 ± 0.01
HCT (%)	40–50	39.21 ± 2.45
MCV (fl)	82–100	93.10 ± 5.865
MCH (pg)	27–34	31.04 ± 2.154
MCHC (g/l)	316–354	332.65 ± 9.509
PDW (%)	10.0–17.9	16.04 ± 1.043
CRP (mg/l)	3.0–10.0	25.09 ± 43.538

**Table 3:** Symptomatic characteristics of all patients

Symptoms	*n*=208 (%)
Fever	146 (70.2)
Nasal congestion	7 (3.4)
Cough	115 (55.3)
Fatigue	47 (22.6)
Poor appetite	28 (13.5)
Dyspnea	57 (27.4)
Diarrhea	9 (4.3)

In total, 53 patients presented with various comorbidities while 155 patients had no noted comorbid conditions (Table 4). In addition, the present study found a statistical difference between comorbid groups for these five laboratory parameters: RBC (*P*=0.001), HGB (*P*=0.004), HCT (*P*=0.01), MCV (*P*=0.01), and CRP (*P*=0.027).

**Table 4:** Comorbid groups characteristics

Laboratory parameters	All patients (*n*=208)	Comorbid		*P*-value
		Without (*n*=155)	With (*n*=53)	
WBC (×10^9^/l)	5.76 ± 2.656	5.76 ± 2.539	5.73 ± 2.999	0.943
RBC (×10^12^/l)	4.24 ± 0.619	4.32 ± 0.612	3.99 ± 0.577	0.001
HGB (g/l)	131 ± 18.255	133.12 ± 18.58	124.84 ± 15.877	0.004
PLTs (%)	190.52 ± 79.492	195.03 ± 80.68	177.18 ± 75.059	0.158
Neutrophils (×10^9^/l)	3.76 ± 2.450	3.73 ± 2.436	3.83 ± 2.436	0.796
Lymphocytes (×10^9^/l)	1.47 ± 0.622	1.51 ± 0.605	1.35 ± 0.664	0.114
Monocytes (×10^9^/l)	0.4 ± 1.99	0.4 ± 0.179	0.4 ± 0.252	0.891
Eosinophils (×10^9^/l)	0.09 ± 0.118	0.09 ± 0.117	0.89 ± 0.123	0.847
Basophils (×10^9^/l)	0.03 ± 0.01	0.45 ± 0.287	0.0 ± 0.011	0.365
HCT (%)	39.21 ± 2.45	39.81 ± 6.11	37.45 ± 4.65	0.01
MCV (fl)	93.10 ± 5.865	92.49 ± 5.728	94.89 ± 5.949	0.01
MCH (pg)	31.04 ± 2.154	30.89 ± 2.134	31.49 ±2.17	0.077
MCHC (g/l)	332.65 ± 9.509	332.91 ± 10.023	331.925 ± 7.854	0.516
PDW	16.04 ± 1.043	16.0 ± 1.147	16.17 ± 0.637	0.293
CRP (mg/l)	25.09 ± 43.538	21.20 ± 42.549	36.47 ± 44.80	0.027

Based on gender characteristics, CRP levels were not significant (*P*=0.155) (Table 5); however, there was a statistical difference in RBC, HGB, neutrophils, monocytes, HCT, MCV, MCH, MCHC, and PDW levels between gender groups.

**Table 5:** Gender groups’ characteristics

Laboratory parameters	All patients (*n*=208)	Gender		*P*-value
		Female (*n*=101)	Male (*n*=107)	
WBC (×10^9^/l)	5.76 ± 2.656	5.31 ± 2.518	6.18 ± 2.72	0.483
RBC (×10^12^/l)	4.24 ± 0.619	4.02 ± 0.466	4.44 ± 0.677	<0.001
HGB (g/l)	131 ± 18.255	122.61 ± 13.032	138.95 ± 18.965	<0.001
PLTs (%)	190.52 ± 79.492	193.82 ± 74.817	187.41 ± 83.899	0.562
Neutrophils (×10^9^/l)	3.76 ± 2.450	3.37 ± 2.415	4.13 ± 2.437	0.0026
Lymphocytes (×10^9^/l)	1.47 ± 0.622	1.48 ± 0.611	1.45 ± 0.635	0.682
Monocytes (×10^9^/l)	0.4 ± 1.99	0.35 ± 0.140	0.45 ± 0.234	0.001
Eosinophils (×10^9^/l)	0.09 ± 0.118	0.89 ± 0.137	0.95 ± 0.986	0.711
Basophils (×10^9^/l)	0.03 ± 0.01	0.12 ± 0.013	0.58 ± 0.345	0.189
HCT (%)	39.21 ± 2.45	36.76 ± 5.180	41.53 ± 5.53	<0.001
MCV (fl)	93.10 ± 5.865	92.26 ± 5.306	93.9 ± 6.268	0.043
MCH (pg)	31.04 ± 2.154	30.53 ± 2.129	31.52 ±2.075	0.001
MCHC (g/l)	332.65 ± 9.509	330.53 ±0 9.235	334.66 ± 9.368	0.002
PDW	16.04 ± 1.043	15.87 ± 1.228	16.20 ± 0.802	0.019
CRP (mg/l)	25.09 ± 43.538	20.67 ± 42.532	29.27 ± 44.259	0.155

## Discussion

The present study included 208 mild and common COVID-19 patients with an almost 1:1 gender ratio. Although 13.5% (*n*=28) of these patients were asymptomatic, the most commonly observed symptoms were fever (*n*=146), cough (*n*=115), fatigue (*n*=47), and dyspnea (*n*=57). Less than 5% of patients presented with nasal congestion (*n*=7), and diarrhea (*n*=9). This research reported mild and common cases compared with Huang et al. (*n*=41) study which observed similar symptoms such as fever (70 vs 98%), cough (55.3 vs 76%), and fatigue (22.6 vs 44%) in more serious and fatal patients. The mild and common patients only presented with few common symptoms such as fever, cough, or fatigue; as a result, the proportion of common symptoms in the present study was low. In addition, we reported some minor symptoms such as poor appetite (13.5%) and nasal congestion (3.4%) in mild and common patients with a relatively high proportion as compared with Huang et al. [7].

The median age of our study was 50.0 years. Several studies have reported an older median age of 50.0–57 years in patients with severe conditions [5,7,8].

The CBC findings of this investigation showed high levels of CRP (25.09 ± 43.538) (Table 4). This rise of CRP can be explained by the response of the human body to the new COVID-19 infection. Our analysis has found lower HGB (124.84 ± 15.877) and reduced HCT (37.45 ± 4.685) and slightly lower RBC (3.19 ± 0.577) levels in patients with comorbid conditions. In addition, there was a significant difference of RBC, HGB, HCT, MCV, and CRP levels between the two comorbid groups with the following respective *P*-values: (*P*=0.001; *P*=0.004; *P*=0.01; *P*=0.01; and *P*=0.027). Hypertension (*n*=41), coronary heart disease (*n*=11), diabetes mellitus (*n*=13), and respiratory diseases (*n*=16) were the different comorbidities found in the present study. The abnormalities of HGB, HCT, and RBC or anemia observed in patients with comorbidities are explained by the inability of the bone marrow to produce enough RBCs to carry oxygen and due to lung damages induced by the COVID-19 which makes gaseous exchange difficult. These abnormalities explained the symptoms of fatigue (22.6%) and dyspnea (27.4%) observed in the population of the present study. On the other hand, the presence of comorbid conditions of these patients might interfere with RBC production due to existing inflammation. All 208 reported cases in this retrospective observational study were not severe. Zhang et al. reported that increased leukocytes (*P*=0.003) were commonly observed in severe cases [5]. In contrast to our observations, no leukocytosis was observed even in the groups presenting with comorbidities. Additionally, the high CRP levels observed in our comorbidity group were inferior compared with Zhang et al. severe patients’ group (CRP 36.47 vs 47.6). The level of CRP was high in patients with severe conditions because of the degree of inflammation, or the COVID-19 infection, or tissue damage. It is also well known that the CRP levels rise during infections [9], and as part of inflammatory processes [10]. This research also found a statistically not significant *P*-value (*P*=0.155) at CRP levels based on gender groups compared with significant comorbidity groups; demonstrating, therefore, that the inflammation is not related to gender. We also observed a statistical difference in RBC, HGB, neutrophils, monocytes, HCT, MCV, MCH, MCHC, and PDW levels between gender groups maybe because these parameters are low in females and RBCs are more easily affected due to infections in females than males; however, the difference of RBC, HGB, HCT, and MCV observed between the two comorbid groups might be related to the virus action against RBC production or destruction or the presence of comorbidities.

Our study also included 13.5% (*n*=28) asymptomatic mild and common patients which were positive to COVID-19 as compared with several studies. This explained that patients infected with this new virus may not show symptoms and that the infection may only be detected by the use of RT-PCR. Given the number of asymptomatic cases (*n*=28) reported in this observational cohort, it is therefore important to all clinicians to consider this factor during diagnosis.

This research does not show any decreased levels of lymphocytes; however, Chen et al. analyzed 29 patients with COVID-19 and reported an increased level of CRP (*n*=27/29) and (*n*=20/29) lymphocytopenia [11]. Another study of nine patients also reported lymphopenia [12]. One study (*n*=138) has recently reported total depressed lymphocyte levels which are similar to findings with SARS-CoV and MERS-CoV. Besides, the same study also found persistent lymphocytopenia and neutrophilia until death [13]. Huang et al. (*n*=41) also reported CBC abnormality such as lymphopenia (63%) [7]. Li et al. have recently shown in one-single arm meta-analysis of 1994 patients a blood count finding of increased CRP (44.3%), leukocytopenia (29.4%), and lymphocytopenia (64.5%) [14]. Laboratory data analysis of 452 patients by Qin et al. showed patients with higher leukocytes count and lower percentages of monocytes, basophils, and eosinophils, as well as lymphocytopenia in the more severe cases [15].

## Conclusion

In summary, the present study has shown the CBCs of 208 mild and common COVID-19 cases and the most likely laboratory findings in these patients were abnormalities in RBCs, HGB, HCT, and CRP. Clinicians should consider these parameters when reading the CBC of COVID-19 patients.

## Abbreviations

CBC: complete blood count
COVID-19: 2019 coronavirus disease
CRP: C-reactive protein
HCT: hematocrit
HGB: hemoglobin
MCHC: Mean corpuscular hemoglobin concentration
MCV: mean corpuscular volume
MERS-CoV: Middle East respiratory syndrome coronavirus
PDW: platelet distribution width
RBC: red blood cell
RT-PCR: reverse transcription-polymerase chain reaction
SARS-CoV: severe acute respiratory syndrome coronavirus
